# Direct observation of multistep energy transfer in LHCII with fifth-order 3D electronic spectroscopy

**DOI:** 10.1038/ncomms8914

**Published:** 2015-07-31

**Authors:** Zhengyang Zhang, Petar H. Lambrev, Kym L. Wells, Győző Garab, Howe-Siang Tan

**Affiliations:** 1 Division of Chemistry and Biological Chemistry, School of Physical and Mathematical Sciences, Nanyang Technological University, 21 Nanyang Link, Singapore 637371; 2 Biological Research Centre, Hungarian Academy of Sciences, Temesvári krt. 62, Szeged 6726, Hungary

## Abstract

During photosynthesis, sunlight is efficiently captured by light-harvesting complexes, and the excitation energy is then funneled towards the reaction centre. These photosynthetic excitation energy transfer (EET) pathways are complex and proceed in a multistep fashion. Ultrafast two-dimensional electronic spectroscopy (2DES) is an important tool to study EET processes in photosynthetic complexes. However, the multistep EET processes can only be indirectly inferred by correlating different cross peaks from a series of 2DES spectra. Here we directly observe multistep EET processes in LHCII using ultrafast fifth-order three-dimensional electronic spectroscopy (3DES). We measure cross peaks in 3DES spectra of LHCII that directly indicate energy transfer from excitons in the chlorophyll *b* (Chl *b*) manifold to the low-energy level chlorophyll *a* (Chl *a*) via mid-level Chl *a* energy states. This new spectroscopic technique allows scientists to move a step towards mapping the complete complex EET processes in photosynthetic systems.

## Abstract (web summary)

Photosynthesis is a complex process, involving the transfer of sunlight driven excitation energy to a reaction centre. Here, the authors directly observe the multistep excitation energy transitions in a light-harvesting complex using ultrafast fifth-order three-dimensional electronic spectroscopy.

## Graphical abstract

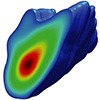

A 2D electronic spectrum is presented in two frequency dimensions, which can be termed the excitation frequency and the emission frequency. Two-dimensional electronic spectroscopy (2DES)^1^,^2^,^3^ measures a two-point correlation plot, and is an excellent tool to measure single-step excitation energy transfer (EET) processes as the appearance and evolution of off-diagonal cross peaks provide a direct observation of electronic coupling between the donor and acceptor pigment molecules, by reading off the corresponding frequencies on the excitation and emission axes, respectively^4^,^5^,^6^,^7^. However, multistep EET processes are only indirectly inferred from the 2DES measurements^8^. Since it is clear that complex multistep EET network is present in most light-harvesting complexes^9^,^10^,^11^,^12^, higher-order multidimensional electronic spectroscopy will be needed to directly measure multistep EET processes.

Fifth-order 3D optical spectroscopy is an extension of third-order 2D optical spectroscopy^13^,^14^,^15^,^16^,^17^,^18^. Applications include the probing of higher states of vibrational systems in order to better characterize the anharmonicity 14,19, the testing of non-Markovian processes^20^ and the characterizing of electron transfer systems^21^.

In fifth-order 3D optical spectroscopy, a spectrum *S*^(5)^(ω_1_, ω_3_, ω_5_; *t*_2_*, t*_4_) with three frequency axes parametrized with two population times *t*_2_ and *t*_4_, is measured. A two-step EET process that proceeds from state A to state B over time *t*_2_ and from state B to state C over time *t*_4_ results in the emergence of a 3D cross peak (ω_1_, ω_3_, ω_5_)=(ω_A_, ω_B_, ω_C_) on a 3D spectrum, where ω_A_, ω_B_ and ω_C_ are the absorption frequencies of state A, B and C, respectively. In comparison, a conventional 2D spectrum is presented as *S*^(3)^ (ω_1_, ω_3_; *t*_2_) with a population time *t*_2._ In addition, if the conventional 2D spectrum *S*^(3)^(ω_1_, ω_3_; *t*_2_) is measured for a series of population times *t*_2_ and Fourier transformed about *t*_2_, one can obtain a data set *S*^(3)^(ω_1_, ω_3_; ω_2_). As there are three frequency axes involved, it has been commonly termed 3D spectroscopy as well^22^,^23^. This third-order 3D optical spectroscopy *S*^(3)^(ω_1_, ω_3_; ω_2_) is different from the fifth-order 3D optical spectroscopy *S*^(5)^(ω_1_, ω_3_, ω_5_; *t*_2_*, t*_4_), which is the subject of this article.

There are several other fifth-order multipulse nonlinear optical experiments such as the family of pump-dump-probe (PDP) and pump-repump-probe spectroscopies^24^ and the multiple population-period transient spectroscopy^25^. In these experiments, the system interacts at two moments in time (separated by a first population period) with the light field. This is followed by a second population period before a transient absorption is obtained. Therefore these experiments give a transient spectrum as a function of the two population time *S*^(5)^(ω_5_; *t*_2_, *t*_4_). There is no frequency resolution (apart from the coarse resolution determined by the laser-pulse spectral bandwidth) for the two interactions. The fifth-order 3D optical spectroscopy described in this paper can be viewed as a general form of these fifth-order experiments. In addition to the two controllable population periods, fifth-order 3D optical spectroscopy has three coherence periods that give rise to three frequency dimensions. This gives the three-point frequency–frequency correlation as a function of two population times *S*^(5)^(ω_1_, ω_3_, ω_5_; *t*_2_*, t*_4_). Another kind of fifth-order optical experiment, 2D Raman spectroscopy was plagued with spurious signal arising from third-order cascading processes^26^. It has been shown in control tests performed for fifth-order resonant optical experiments (such as the fifth-order 3D electronic spectroscopy experiments discussed in this article) that there is no significant contamination from third-order cascading signals^15^,^19^,^27^. If for some systems or conditions that the cascading signals become significant, procedures are available to isolate the contaminant signals^28^.

A fifth-order 3D optical spectroscopy experiment involves the control of up to six (including local oscillator pulse) phase-locked pulses and is extremely challenging. Purely absorptive fifth-order 3D optical spectroscopy was first demonstrated in the mid-infrared^29^ and only recently did we first demonstrate a purely absorptive fifth-order 3D electronic spectroscopy in the visible (three-dimensional electronic spectroscopy (3DES)) based on a pulse shaping pump-probe setup^17^,^27^.

Plant light-harvesting complex II (LHCII)—the most abundant membrane protein in nature, is responsible for the absorption of about half of the radiation used for plant photosynthesis and plays key roles in regulating the energy input to Photosystem II to optimize photosynthetic efficiency while avoiding damage^30^,^31^.

In this article, we report the use of 3DES to directly observe two-step EET processes in LHCII. We observe 3D cross peaks that represent a two-step EET process from the chlorophyll *b* (Chl *b*) manifold to the low-energy level chlorophyll *a* (Chl *a*) via mid-level Chl *a* energy states. The population time dependence of the 3D cross peak amplitude corresponds well with corresponding 2DES measurements. We provide possible assignments of the observed spectral features based on published structure-based models.

## Results

### Fifth-order 3DES

**Figure 1:**
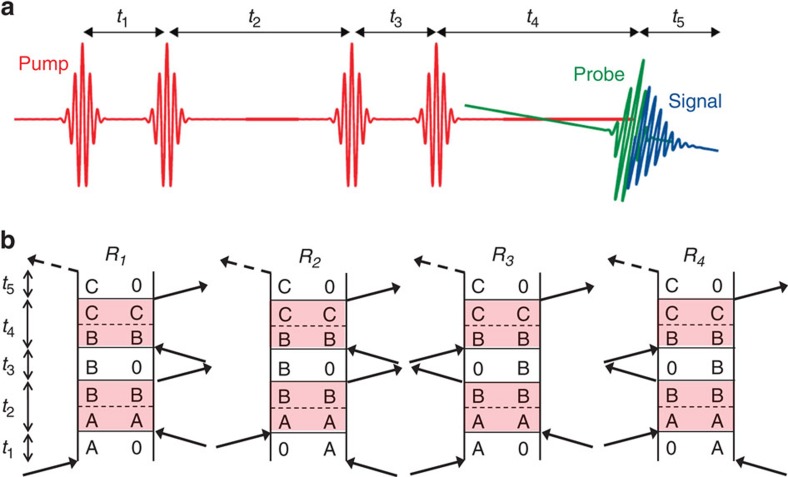
Three-dimensional electronic spectroscopy (3DES) pulse sequence and Liouville pathways diagrams. (**a**) Pulse sequence used in coherent fifth-order 3D optical spectroscopy performed in a pulse-shaper assisted pump-probe geometry. The pulse-shaper creates a four-pulse pump sequence (red) with controllable delays and relative phases followed by the probe (green) that interacts at a small angle to emit the signal (blue). (**b**) A 3D cross peak (ω_1_, ω_3_, ω_5_)=(ω_A_, ω_B_, ω_C_) on a 3DES spectrum is a combination of fifth-order optical processes outlined in the Liouville pathways (double-sided Feynman diagrams) depicted above^16^. The shaded areas denote the population periods where EET processes proceed between the states separated by the dashed lines. The label of the processes *R*_1_, *R*_2_, *R*_3_ and *R*_4_ follows the convention of Hamm^10^.

In fifth-order 3DES, five laser pulses, with inter-pulse delay times *t*_1_, *t*_2_, *t*_3_ and *t*_4_, interact with the sample and a fifth-order optical signal is emitted at time *t*_5_. The pulse sequence scheme is shown in Fig. 1a. The periods *t*_1_, *t*_3_ and *t*_5_ are the coherence times where the excitons oscillate at their transition frequencies. The periods *t*_2_ and *t*_4_ are the population times, during which the population transfers between different excitons. The final coherence over *t*_5_ results in an optical signal that is emitted in a phase-matched direction and can be measured by frequency-resolved heterodyne detection (along the ω_5_ axis). At given population times *t*_2_ and *t*_4_, the experiment is scanned over the coherence periods *t*_1_ and *t*_3_. The data are processed and then Fourier transformed about coherence times *t*_1_ and *t*_3_ to obtain a 3D spectrum *S*^(5)^(ω_1_, ω_3_, ω_5_; *t*_2_*, t*_4_). The 3D cross peak feature that represents a two-step EET process that proceeds from state A to state B over time *t*_2_ and from state B to state C over time *t*_4_, can be outlined using the Liouville pathways diagrams (double-sided Feynmann diagrams)^32^ in Fig. 1b. The labels for the processes *R*_1_, *R*_2_, *R*_3_ and *R*_4_ follow the convention of Hamm^13^. The shaded areas denote the population periods where EET processes proceed between the states separated by the dashed lines.

### Linear spectroscopy and 2DES of LHCII

**Figure 2:**
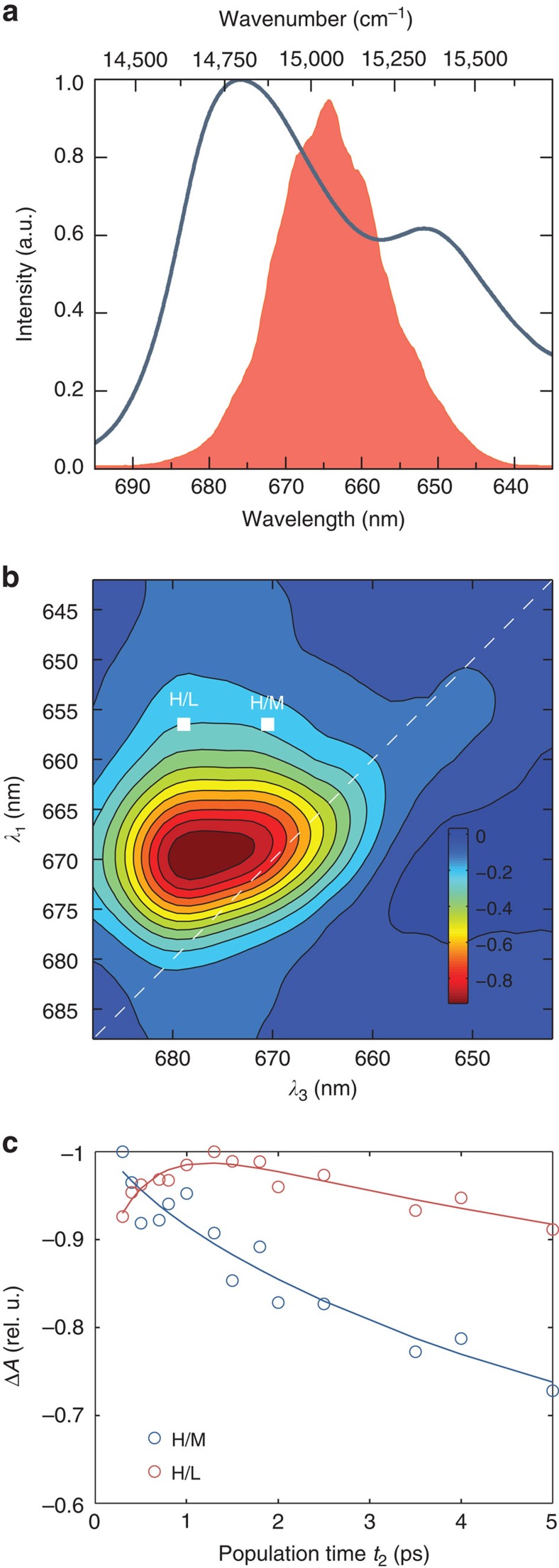
Linear and 2DES spectrum of LHCII. (**a**) The experimental linear absorption spectrum of LHCII (solid blue) and the excitation pump spectrum (solid filled pink). (**b**) Two-dimensional spectrum of LHCII at population time *t*_2_=0.3 ps, with the white line indicating the diagonal of the spectra. (**c**) The amplitude evolution of the integrated exciton cross peaks 657→670 nm and 657→678 nm as a function of population time t_2_. A multistep EET process can be inferred from the associated decay of the 657→670 nm cross peak and the rise of the 657→678 nm cross peak.

According to the crystallographic structure of LHCII^33^, each monomer in the trimeric pigment–protein complex binds 14 chlorophylls (8 Chl *a* and 6 Chl *b*^33^). The 14 excitons are closely spaced, which makes it hard to elucidate the different timescales of every energy migration dynamics in the multistep EET processes. Henceforth we will adopt the more often used wavelength unit, λ instead of the frequency unit ω. The linear absorption spectrum (Fig. 2a) can be divided into three regions: high-energy exciton region (λ_H_<660 nm), encompassing transitions to Chl *b* and high-energy Chl *a* exciton states, the mid-energy region (λ_M_=660–675 nm), which contains mid-energy Chl *a* states, and the low-energy exciton region (λ_L_>675 nm), which contains the lowest-energy Chl *a* exciton states. In the following, we will demonstrate how 3DES can be employed to observe the multistep flow of energy between the three wavelength regions λ_H_→λ_M_→λ_L_.

As a reference, 2DES of the EET process was performed as well, and a representative 2D spectrum at population time *t*_2_=300 fs is shown in Fig. 2b. More 2D spectra at various *t*_2_ have been included in Supplementary Fig. 1. Two cross peak regions, H/L and H/M, are labelled in Fig. 2b, which is ascribed to EET from the high-energy exciton region to the low-energy region (λ_H_→λ_L_) and to the mid-energy region (λ_H_→λ_M_), respectively. The cross-peak amplitudes at H/L (655→678 nm) and H/M (655→670 nm) are plotted against *t*_2_ in Fig. 2c. The H/M amplitude initially rises rapidly on a timescale of ∼300 fs due to EET from Chls *b* to mid-energy Chls *a*. The H/M cross peak subsequently decays on a longer timescale of ∼2 ps, which is a result of EET out of the mid-energy Chl *a* region. The H/L cross peak amplitude shows only an increasing trend. Apart from indicating the direct λ_H_→λ_L_ transfer, a multistep EET process λ_H_→λ_M_→λ_L_ can be indirectly inferred from the correlated timescale of the decay of the H/M peak and the rise of the H/L cross peak, as it has been previously reported^8^,^34^. It is evident that the cross peaks may not be readily resolved in the 2D spectra, due to the broad inhomogeneous widths of the spectral features. In this regard, 3DES has the advantage of being able to spectrally resolve closely spaced or overlapping exciton bands in the additional frequency axis ω_5_, and also by correlating energy transfers with the three frequency axes.

### Fifth-order 3DES of LHCII

**Figure 3:**
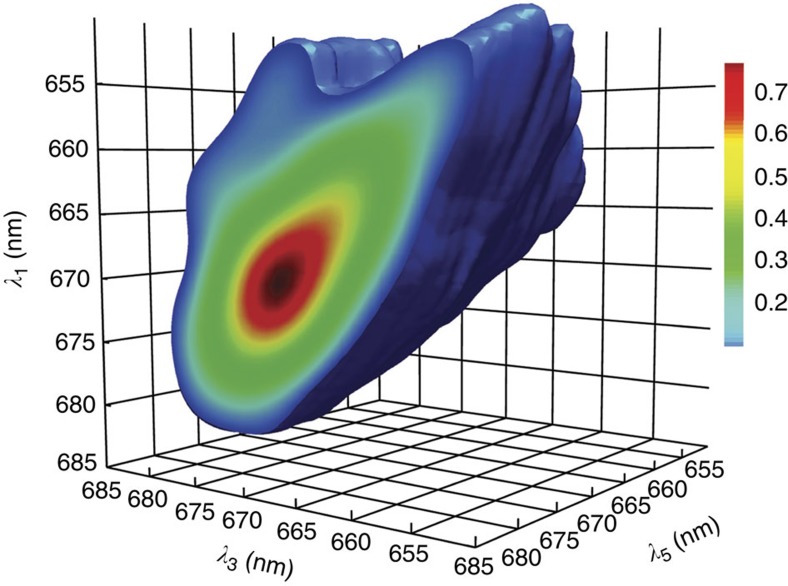
Three-dimensional electronic spectroscopy (3DES) spectrum of LHCII. Spectrum recorded at population times *t*_2_=0.3 ps and *t*_4_=800 fs. The isosurface represents amplitude values of 0.1 relative to the global maximum. The cutaway at λ_5_=685 nm to better illustrate the features and structure of the 3D spectra. The prominent feature to note is the ridge along the λ_5_ axis, around λ_1_=655, λ_3_=670. The ridge's shape and feature evolves with the different population times *t*_4_ in our experiments.

In the 3D spectra of LHCII presented in this article, the population time *t*_2_ was kept fixed at 300 fs, and spectra were taken at different population times *t*_4_ from 150 fs to 8 ps. A graphical representation of the 3D spectrum at *t*_2_=300 fs, *t*_4_=800 fs is shown in Fig. 3. The rendered isosurface represents amplitude values of 0.1 relative to the global maximum. The maximum along λ_1_ and λ_3_ is found near the excitation wavelength, 668 nm; the peak along the detection wavelength λ_5_ coincides with the pump-probe maximum, 678 nm. A cutaway is made at λ_5_=682 nm to better illustrate the features and structure of the 3D spectra. The amplitude of the 3D spectrum on this cutaway slice is colour coded. The amplitude is concentrated on the λ_3_=λ_1_ diagonal plane, representing all the states whose population has not decayed during *t*_2_. It is evident from the figure that the 3D spectrum features additional cross-correlation amplitudes off the λ_3_=λ_1_ diagonal plane, particularly in the region λ_3_<λ_1._ This indicates downhill energy transfer during the population time *t*_2_. One prominent feature to note is a ridge (or 3D cross peak) along the λ_5_ axis, around λ_1_=655 nm, λ_3_=670 nm. The ridge's shape and feature evolves with the different population times *t*_4_ in our experiments and is the main subject of our discussion below. For comparison, representative 3D spectra at *t*_2_=300 fs and *t*_4_=150 fs and 5 ps are presented in Supplementary Fig. 2. Qualitatively, one can discern the evolution of the 3D cross peaks clearly with different delays *t*_4_.

**Figure 4:**
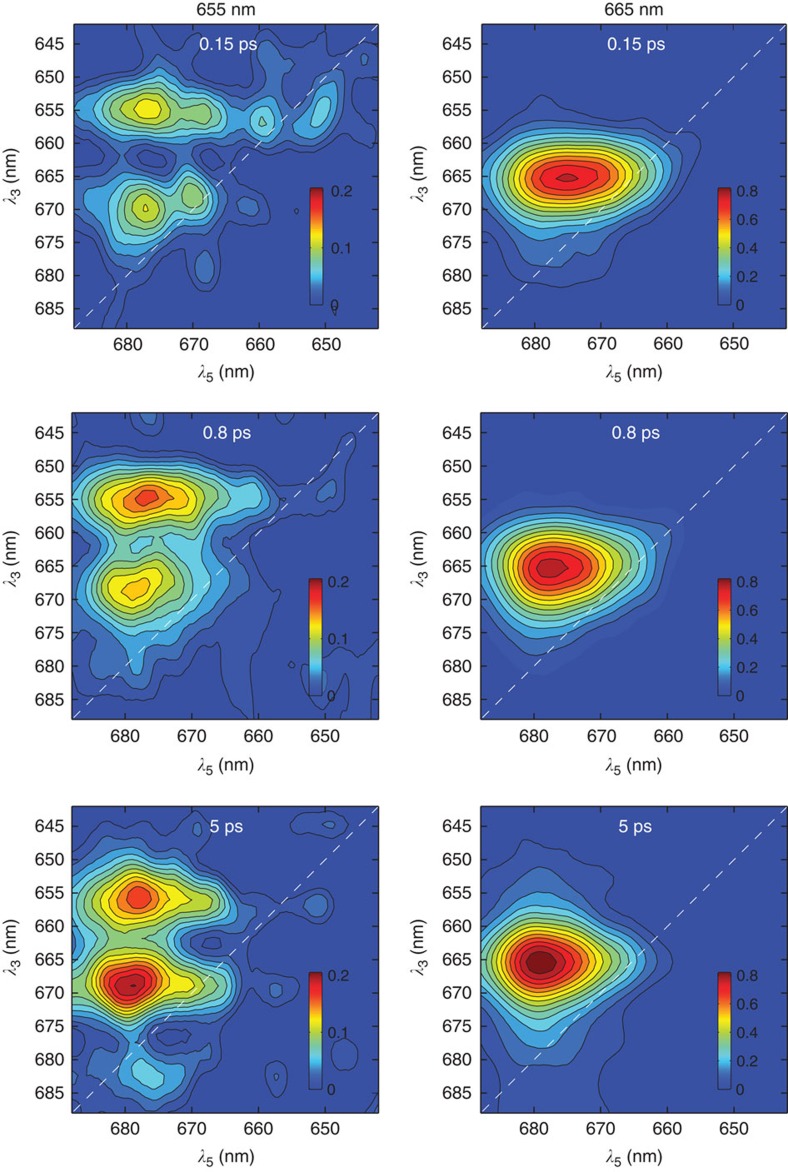
Two-dimensional slices of 3DES spectra. Slices of the 3D spectra of LHCII at λ_1_=655 nm and λ_1_=665 nm to obtain quasi-2D spectra, with selected quasi-2D spectra shown at population times *t*_2_=0.3 ps and *t*_4_=0.2, 0.8 and 5 ps (indicated on the plots). The colour scale represents amplitudes relative to the respective 3D spectrum's global maximum.

To better visualize the time evolution of the 3D cross peaks, slices of the 3D spectra at wavelengths λ_1_=655 nm and λ_1_=665 nm for three different population times *t*_4_ are shown as quasi-2D spectra with wavelength axes λ_3_ and λ_5_ (Fig. 4). The spectra demonstrate the potential of 3DES to selectively probe EET pathways with high resolving power. The slices at λ_1_=655 nm feature two well-separated cross peaks, at λ_3_∼655 nm and λ_3_∼670 nm, whereas the slices at λ_1_=665 nm have only one band around 665 nm. The most significant feature on the presented slices is the 3D cross peak 655→670→678 nm, which can be denoted H/M/L, and interpreted as follows. In a 2D spectrum at population time *t*_2_, the H/L cross peak amplitude is the conditional population of state L (absorbing at λ_L_) after population time *t*_2_, given that the initial excitation is at H (absorbing at λ_H_). Extending the interpretation to 3DES, the 3D cross peak amplitude at H/M/L is the conditional population of L after population time *t*_2_+*t*_4_ given that (i) the initial excitation is at H (λ_H_∼655 nm) and (ii) the population after time *t*_2_ is at M (λ_M_∼670 nm). This gives a direct measurement of a two-step EET process between three different states: H→M→L. The other features of interest in the spectra are along λ_1_=655 nm, that is, on the diagonal plane λ_3_=λ_1_, denoting EET from states H to states L (H/H/L cross peak) and from H to M (H/H/M cross peak).

**Figure 5:**
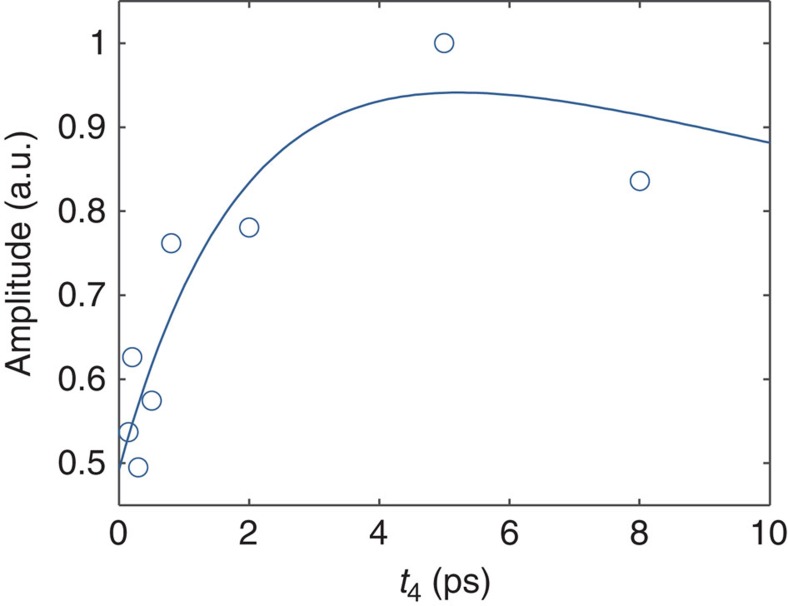
Population time dependence of 3D cross peak amplitude. Experimental values (blue circles) and simulation trace (blue line) of the H/M/L cross peak amplitudes in the quasi-2D spectra (λ_1_=656 nm slice; *t*_2_=0.3 ps) as a function of the second population time *t*_4_.

Owing to the separation over a third frequency (wavelength) dimension, the spectral regions H and M are fully resolved in the 3D spectra, whereas in the 2D spectra (Fig. 2b) they overlap. Moreover, by virtue of using two population times, the kinetics of the direct H→L and multistep H→M→L pathways can be unambiguously resolved. The rise dynamics of the H/H/L cross peak amplitude depends solely on the H→L process, and the rise of the H/M/L peak depends solely on the M→L process, allowing us, in principle, to determine the respective rate constants directly from the experimental data without the need for soft modelling and the inherent uncertainty associated with it. Figure 5 plots the experimental amplitudes of the H/M/L cross peak as a function of population times *t*_4_. We simulate the H/M/L cross peak amplitudes by solving a rate equation for a system comprising H, M and L states (Supplementary Note 2). As parameters for the simulations, we use the EET transfer timescales values that have been recently obtained for LHC II trimers^34^, where the Chl *b* excitonic states (associated with the H states here) transfer energy to Chl *a* states (associated with the M and L states here) at a timescale of 300 fs and the intermediate Chl *a* states (associated with the M states here) to low-energy Chl *a* states (associated with the L states here) at a 2.3 ps timescale. There is then a >20 ps relaxation to ground state g. The simulated values (red line in Fig. 5) are consistent with the trend of the experimental values, as can be seen in Fig. 5. The line essentially traces a 2.3 ps timescale rise that represents the M→L transfer and a subsequent >20 ps decay representing the decay to ground state.

## Discussion

Assignment of spectral features to structural moieties in the pigment–protein complex is possible by use of structure-based models of the exciton energies and transfer kinetics^9^,^10^,^12^,^35^,^36^. On the basis of more recent model by Müh *et al*.^36^, the H state (λ_H_∼655 nm) can be contributed by either Chl 608 (∼658 nm) in the stromal layer of Chls *b*, or Chl 607 (∼654 nm), in the lumenal Chl *b* cluster. Although both states are within the relevant wavelength, Chl 607 is incompatible with the observed H/H/L cross peak at *t*_2_=300 fs. EET from this Chl *b*, on the lumenal side, to the L state, which is identified with the Chl 610/611/612 cluster on the stromal side, must occur over a much longer time scale. In contrast, rapid EET (≤1 ps) from Chl 608/609 to the 610/611/612 cluster, is expected by the structural models of Novoderezhkin *et. al*.^10^,^12^, which is in line with the dynamics of the 3D cross peak. As to the identity of the M state (λ_M_∼670 nm), several pigments may contribute to this spectral region but only the Chl 602/603 pair (also according to Novoderezhkin *et al*.^10^) would have the longer lifetime compatible with the 3DES data. The available structural models provide different time constants for EET depending on the chosen theoretical formalism. The EET from Chl 602/603 to the lowest-energy Chls is estimated to be from subpicoseconds^10^ to tens of picoseconds^12^. The kinetics of the H/M/L peak in our experiment is consistent with the rate constant of M→L transfer between 2 and 6 ps which falls closer to the more recent calculation by Renger *et al*.^12^

The results presented in this report demonstrate for the first time that direct observation of multistep EET processes in LHCII can be achieved using 3DES. The method allows us to selectively probe EET between single exciton states with defined transition energies, isolated from the congested spectrum of the exciton network. In principle, with two independent population times, the two rate constants of a two-step EET process can be directly obtained from experimental data by measuring the population time-dependent amplitudes of appropriate 3D cross peaks. Fifth-order 3DES represents a valuable extension to 2D electronic spectroscopy, which is rapidly becoming a standard tool in photosynthesis research, and will undoubtedly help in unravelling the complex energy transfer processes in biological light-harvesting systems. As a much longer experimental time needed to collect a 3D spectrum compared with a 2D spectrum, it may not be practical to obtain 3D spectra for an exhaustive range of population times *t*_2_ and *t*_4_. It is envisioned that 2DES will first be deployed to obtain as much information as possible about the EET rates. 3DES can then be performed for selected population times *t*_2_ and *t*_4_ to resolve specific exciton states involved in the multistep EET processes.

## Methods

### Sample preparation

The LHCII trimers sample was purified from spinach thylakoid membranes and solubilized with *α*-dodecyl maltoside (*α*-DM) using sucrose-gradient ultracentrifugation^37^. The sample was mixed with nitrogen-flushed 0.06% *α*-DM and 10 mM HEPES buffer solution, and placed in a 1 mm sapphire cell at room temperature. The absorbance of the sample at 674 nm was measured to be 0.2. All linear, 2D and 3D spectra were acquired at room temperature.

### Data acquisition

Our implementation of third-order 2DES^38^ and fifth-order 3DES^17^,^27^ has been described in detail elsewhere. In brief, in the pump-probe geometry^39^,^40^,^41^,^42^ implemented 3DES, the pulse shaper was used to create a four-pulse ‘pump' train (1 kHz laser pulses from an optical parametric amplifier centred at 668 nm. Pulse duration is 43 fs; see Fig. 2a) while a white light continuum pulse is used as the ‘probe' (that provides the fifth interaction). In the present setup, the pulse shaper is able to produce pulse trains whereby the maximum delay between the first and fourth pulses is about 1 ps. Both coherence times *t*_1_ and *t*_3_ were increased in 7 fs time steps from 0 fs to 140 fs. The maximum population time *t*_2_ for our present setup is therefore about 600 fs. The delay for the other population time *t*_4_ is achieved by a conventional optomechanical delay stage, and the maximum achievable *t*_4_ is determined by the length of the delay stage. To obtain the signal from the desired processes (Fig. 1b), phase cycling^43^,^44^,^45^ is necessary. A 3 × 3 × 3 × 1 phase cycling scheme^27^,^45^ was used to obtain the purely absorptive 3D spectra. For the experiments reported in this article, the population time *t*_2_ was set at 300 fs, and population times *t*_4_ were measured at 150, 200, 300, 500, 800, 2,000, 5,000 and 8,000 fs. The 3D spectra were normalized to the maximum intensity of the 2D projection at frequency axis (λ_1_, λ_3_). The pulse shaper was referenced to a carrier frequency of 420 THz and the signal was collected in a partially rotating frame. The ‘probe' beam is measured with a spectrometer (Acton SP2300, Princeton Instruments) and recorded with a 100-pixel × 1,340-pixel charge-coupled device camera (PIXIS 100B, Princeton Instruments) giving the wavelength axis λ_5_. The data is Fourier transformed about *t*_1_ and *t*_3_ to give the frequency axes ω_1_ and ω_3_, which was then converted to wavelength axes λ_1_ and λ_3_. Scatter subtraction and Fourier transformation into the frequency spectrum was performed as reported previously^27^,^38^. For the accompanying 2DES, a two-pulse ‘pump' train with a 3 × 1 phase cycling scheme^38^ is used. The population times *t*_2_ were recorded at a series of delays from 120 fs to 10,000 fs. In 2D optical spectroscopy, a common method to characterize possible experimental artefacts is to apply the 2D projection-slice theorem^39^. In this method, the 2D spectrum is integrated over one frequency axis and compared with the transient absorption spectrum. The 2D projection-slice theorem states that the 2D spectrum should be similar to the 1D spectrum. Analogous to the 2D counterpart, the 3D projection-slice theorem has also been developed^13^. We compare the experimental 3D spectra integrated along one of the frequency axes with the corresponding experimental 2D spectra to verify that our 3D spectra are largely free of experimental artefacts. The details of the comparison are provided in the Supplementary Note 1.

## Additional information

**How to cite this article:** Zhang, Z. *et al*. Direct observation of multistep energy transfer in LHCII with fifth-order 3D electronic spectroscopy. *Nat. Commun.* 6:7914 doi: 10.1038/ncomms8914 (2015).

## Supplementary Material

Supplementary Information Supplementary Figures 1-3, Supplementary Note 1-2 and Supplementary References
