# Supplementary material for: Direct observation of multistep energy transfer in LHCII with fifth-order 3D electronic spectroscopy
Source: Nat Commun. 2015 Jul 31;6:7914. doi: 10.1038/ncomms8914 (PMC4532882; doi:10.1038/ncomms8914)
Supplement: Supplementary Information — Supplementary Figures 1-3, Supplementary Note 1-2 and Supplementary References [file ncomms8914-s1.pdf]

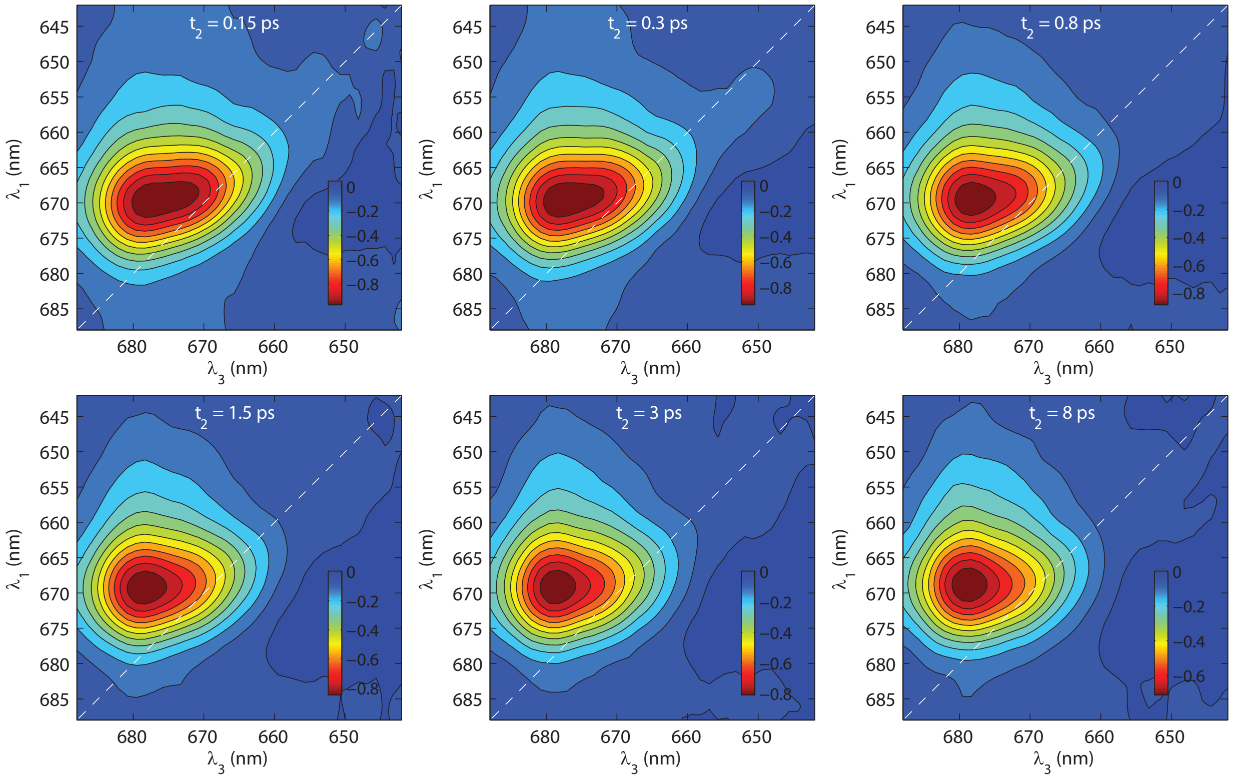

Supplementary Figure 1. 2DES spectra of LHCII. A selection of 2D spectra of LHCII trimers at various population times t_2.

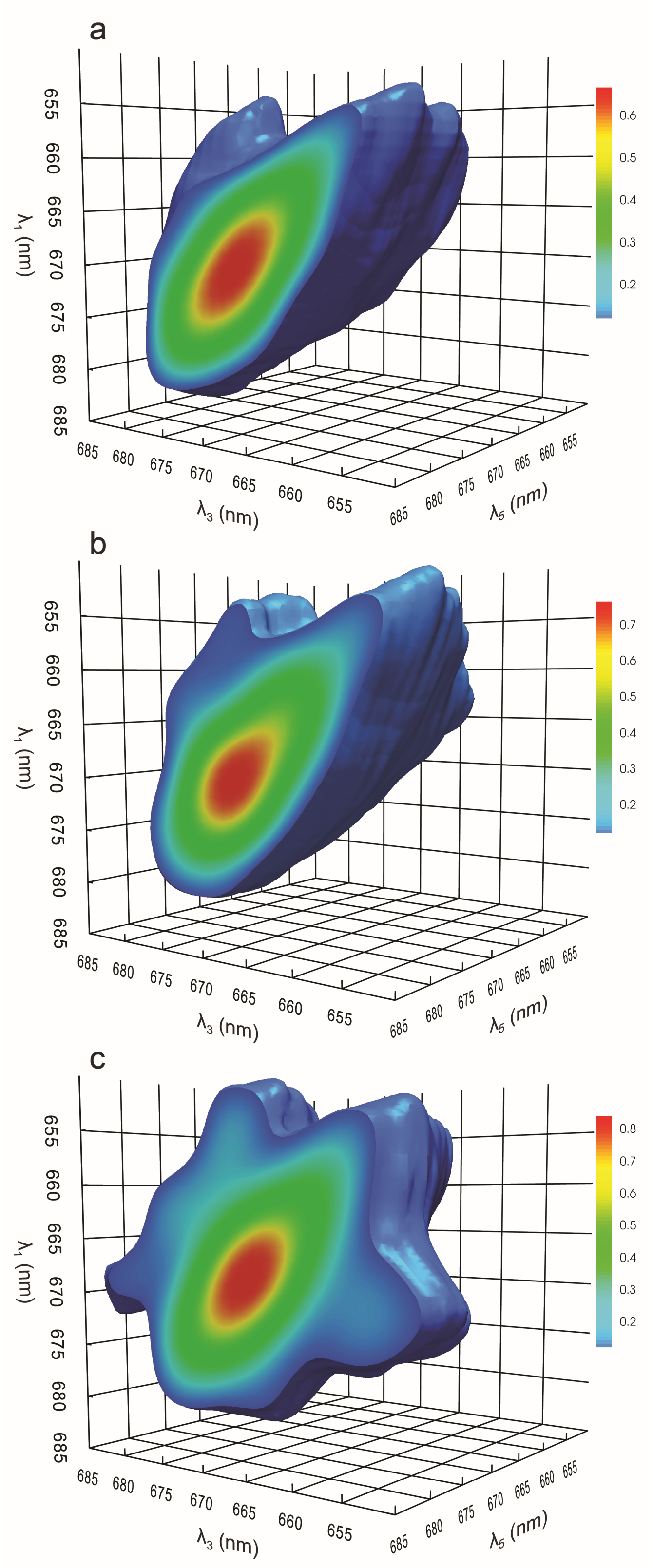

Supplementary Figure 2. 3DES spectra of LHCII. 3D spectra represented by isosurfaces rendered at amplitude values of 0.12 relative to the global maximum for t_2 = 0.3 ps and t_4 = 0.15 ps (a), 0.8 ps (b) and 5 ps (c). The cutaways are made at λ_5 = 682 nm. The prominent ridge (or 3D cross peak) along the λ_5 axis, around (λ_1 = 655 nm, λ_3 = 670 nm) evolves with t_4.

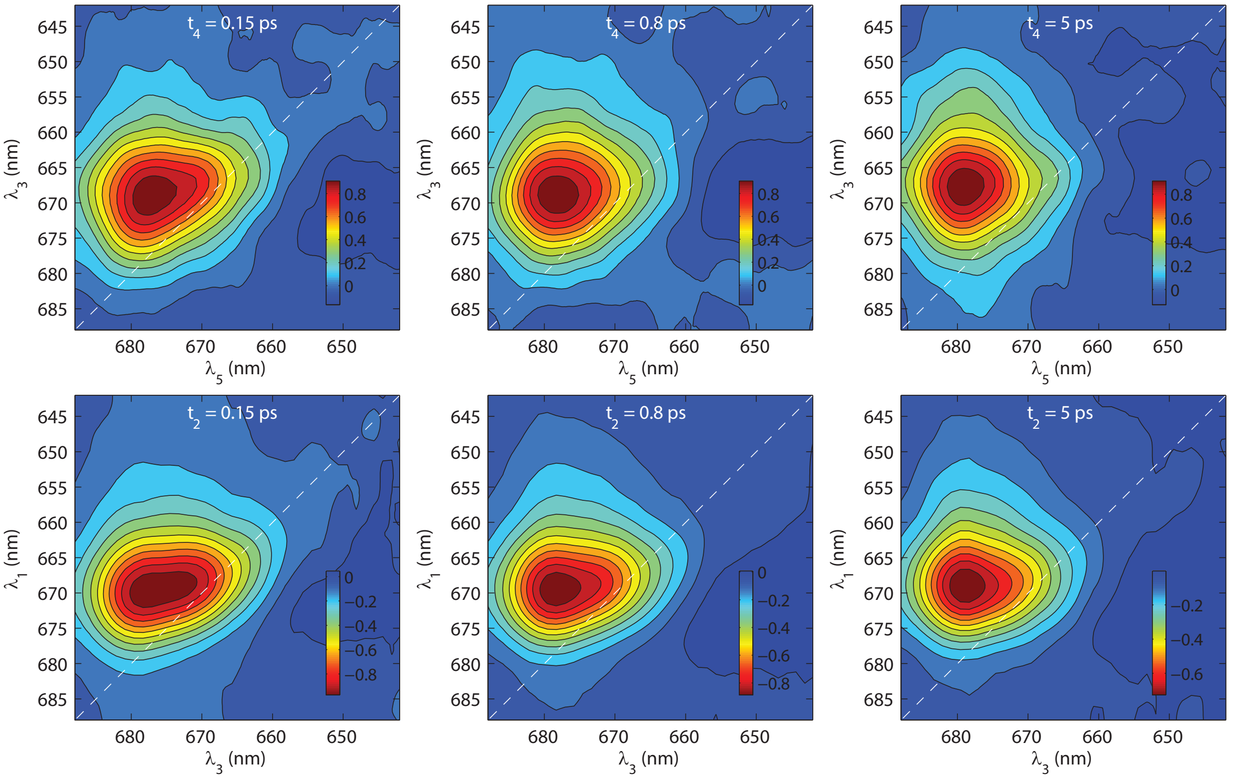

Supplementary Figure 3. Comparison of 2D spectra and projected 3D spectra. Top row: Projected experimental 3D spectra onto 2D performed according to Eq. S.1, for t_4= 0.15 ps, 0.8 ps and 5 ps. Bottom row: Experimental 2D spectra for t_2 = 0.15 ps, 0.8 ps and 5 ps.

Supplementary Note 1. 3D projection slice theorem

The 3D projection slice theorem[1] states that, for a two level system, if we integrate the fifth order 3D spectrum over frequency axis ω_1, we will recover a 2D spectrum:

$$\int_{-\infty}^{\infty} S^{(5)}(\omega_1, \omega_3, \omega_5; t_2, t_4)\, d\omega_1 = S^{(3)}(\omega_3, \omega_5; t_4) \qquad \text{Equation 1}$$

We can take advantage of this relationship to compare the integrated experimental 3D signals $\int_{-\infty}^{\infty} S(\omega_1, \omega_3, \omega_5; t_2, t_4)\, d\omega_1$ at different second population times t_4 with corresponding experimental 2D spectra at population time t_2, $S(\omega_1, \omega_3; t_2)$. A close resemblance between the integrated 3D signals and the 2D spectra is a good indication that the features measured in the 3DES spectra are not artefacts. In the top row of Supplementary Fig. 3 is a series of the projections of 3D spectra (Eq. 1) at t_4 = 150 fs, 800 fs and 5 ps. In the bottom row of Supplementary Fig. 3 is a series of experimental 2D spectra with matching population times t_2. For the 2D spectrum at t_2 = 150 fs, there is still amplitude near the diagonal at λ_1, λ_3 ~ 670 nm. This diagonal feature decays with increasing t_2 as population is transferred to the red part of the spectrum, as is apparent from the subsequent 2D spectra at t_2 = 800 fs and 5 ps. Detailed discussions of these dynamics can be found in our earlier publication[2]. As it is expected, the projection slices of the 3D spectra clearly follow the same trend, viz. the amplitude near the diagonal at λ_1 , λ_3 ~ 670 nm decays with increasing t_4.

We should point out that Eq. 1 is usually applied to a two-level system, i.e. considering only transitions from the ground state to the first electronic excited state $|S_0\rangle \rightarrow |S_1\rangle$. In the case of a real system like LHCII, when the projection of Eq. 1 is performed, we have to include into $R^{(3)}(\omega_3, \omega_5; t_4)$ excited state absorption (ESA) $|S_1\rangle \rightarrow |S_n\rangle$ during the first coherence time. This response $R^{(3)}(\omega_3, \omega_5; t_4)$ is not recovered in typical 2DES spectra, as typical 2DES only recovers ground state absorption transition $|S_0\rangle \rightarrow |S_1\rangle$ over the first coherence time. However, the ESA of chlorophyll is much smaller than that of the absorption transition $|S_0\rangle \rightarrow |S_1\rangle$ [3], hence, as a first approximation, it can be neglected. That is the main reason why in Supplementary Fig. 3, there is close correspondence between the integrated experimental 3D signals and the corresponding experimental 2D spectra.

Supplementary Note 2. Simulation of 3D cross peak amplitude

We present here the analysis and simulation of the 3D cross peak amplitudes. The amplitude of the 3D crosspeak λ_L/ λ_M / λ_L, is the conditional population of excited states absorbing at wavelength λ_L after population time $t_2 + t_4$, given that the initial excitation is at λ_H, and that after population time t_2 the excitation is at λ_M. This conditional amplitude will be denoted henceforth as $A(0, \lambda_H \mid t_2, \lambda_M \mid t_2{+}t_4, \lambda_L)$ To simulate this, we calculate the time-dependent populations of the states H, M, and L by solving a kinetic rate equation

$$\dot{\mathbf{p}}(t) = \mathbf{K}\mathbf{p}(t) \qquad \text{Equation 2}$$

where $\mathbf{p}(t)$ is a vector of time-dependent state populations $\mathbf{p}(\mathrm{t}) = [p_H(t)\ p_M(t)\ p_L(t)]$, respectively, and $\mathbf{K}$ is the matrix of transfer rate constants. The solution of the rate equation can be expressed as

$$\mathbf{p}(t) = \mathbf{M}\, e^{\mathbf{\Lambda} t}\, \mathbf{M}^{-1}\mathbf{p}(0) \qquad \text{Equation 3}$$

where $\mathbf{M}$ and $\mathbf{\Lambda}$ are the eigenvectors and eigenvalues of $\mathbf{K}$, respectively, and $\mathbf{M\Lambda} = \mathbf{KM}$.

To obtain the amplitude of the 3D cross peak λ_H / λ_M / λ_L, $A(0, \lambda_H \mid t_2, \lambda_M \mid t_2{+}t_4, \lambda_L)$, $\mathbf{p}(t_2)$ is first solved using Eq. 3 with the initial population vector $\mathbf{p}(0) = [1\ 0\ 0]$ in order to find the population of state M at time t_2, $p_M(t_2)$. The equation is then solved again with the initial population vector $\mathbf{p}(0) = [0\ p_M(t_2)\ 0]$. The resultant $p_L(t_4)$ is the cross-peak amplitude $A(0, \lambda_H \mid t_2, \lambda_M \mid t_2{+}t_4, \lambda_L)$.

The transfer rate constants used in Eq. 2 are based on the EET transfer timescales values reported recently for LHCII trimers[2], where the Chl *b* excitonic states (associated with the H states here) transfer energy to Chl *a* states (associated with the M and L states here) at a timescale of 300 fs and the intermediate Chl *a* states (associated with the M states here) to low-energy Chl *a* states (associated with the L states here) at a 2.3 ps timescale and >20 ps relaxation to ground state g. $k_{H\to M} = k_{H\to L} = (300\ \mathrm{fs})^{-1}$ and $k_{M\to L} = (2.3\ \mathrm{ps})^{-1}$. We have restricted the rate constants of relaxation from the three excitonic states to the ground state to be the same, $k_g = (20\ \mathrm{ps})^{-1}$. The matrix $\mathbf{K}$ in Eq. 2 in this case will be

$$\mathbf{K} = \begin{pmatrix} -(k_{H\to M} + k_{H\to L} + k_g) & 0 & 0 \\ k_{H\to M} & -(k_{M\to L} + k_g) & 0 \\ k_{H\to L} & k_{M\to L} & -k_g \end{pmatrix}. \qquad \text{Equation 4}$$

Supplementary References

1 Hamm, P. Three-dimensional-IR spectroscopy: Beyond the two-point frequency fluctuation correlation function. *J. Chem. Phys.* **124**, 124506 (2006).

2 Wells, K. L., Lambrev, P. H., Zhang, Z., Garab, G. & Tan, H.-S. Pathways of energy transfer in LHCII revealed by room-temperature 2D electronic spectroscopy. *Phys. Chem. Chem. Phys.* **16**, 11640-11646 (2014).

3 De Boni, L., Correa, D. S., Pavinatto, F. J., dos Santos, D. S. & Mendonca, C. R. Excited state absorption spectrum of chlorophyll a obtained with white-light continuum. *J Chem Phys* **126**, 165102 (2007).
